# Serpin E1 mediates the induction of renal tubular degeneration and premature senescence upon diabetic insult

**DOI:** 10.1038/s41598-023-43411-4

**Published:** 2023-09-27

**Authors:** Bo Han Chen, Xiao Qing Lu, Xian Hui Liang, Pei Wang

**Affiliations:** 1 Blood Purification Center, The First Affiliated Hospital of Zhengzhou University, Zhengzhou, China; 2 Research Institute of Nephrology, Zhengzhou University, Zhengzhou, China; 3 Blood Purification Center, Department of Nephrology, The First Affiliated Hospital of Zhengzhou University, 1 East Jianshe Road, Zhengzhou, 450052 Henan China

**Keywords:** Diseases, Nephrology

## Abstract

As a leading cause of chronic kidney disease, diabetic kidney disease (DKD) involves insidious but progressive impairments of renal tubules, and is associated with premature renal aging. The underlying pathomechanisms remain elusive. Post hoc analyses of the publicly-available renal transcriptome revealed that TGFβ1 is overexpressed in renal tubulointerstitia in patients with DKD and positively correlated with kidney aging signaling. This finding was validated in kidney biopsy specimens collected from patients with DKD, associated with renal tubular senescence and degenerative changes. In vitro in renal tubular epithelial cells, exposure to a diabetic milieu, stimulated with high ambient glucose and TGFβ1, elicited premature senescence, as evidenced by staining for senescence-associated β-galactosidase activity and increased expression of p16^INK4A^, and p53. This coincided with Serpin E1 induction, in parallel with increased fibronectin accumulation and reduced expression of the epithelial marker E-cadherin, all indicative of degenerative changes. Reminiscent of the action of typical senolytics, a small molecule inhibitor of Serpin E1 substantially mitigated the pro-senescent and degenerating effects of the diabetic milieu, suggesting an essential role of Serpin E1 in mediating renal tubular senescence upon diabetic insult. Moreover, inhibition of Serpin E1 abolished the diabetic insult-triggered paracrine senescence of renal tubular cells. In consistency, in patients with DKD, renal tubular expression of Serpin E1 was upregulated and positively correlated with tubular senescence and fibrosis in renal tubulointerstitia. Collectively, diabetic insult induces renal tubular degeneration and premature senescence via, at least in part, Serpin E1 signaling.

## Introduction

As a serious complication of type 1 or type 2 diabetes mellitus, diabetic nephropathy (DN) or diabetic kidney disease (DKD) is one of the leading causes of progressive chronic kidney disease and end-stage renal diseases (ESRD) in Western societies and developed countries^1^. With insidious onsets, DKD is featured by a slow disease course but persistent progression that may last for decades before overt symptoms, such as proteinuria and edema, could be clinically noted^1,2^. For early diagnosis of DKD, microalbuminuria has been widely used in clinical practice, as an indicator of early impairment of the glomerular filtration barrier by DKD, even though it is not necessarily associated with kidney function decline in diabetic patients^3^. In addition to glomerular impairment, the pathology of DKD also involves renal tubular and interstitial injuries, characterized by renal tubular hypertrophy in the early stage, followed by degenerative changes and atrophy of renal tubules in advanced stages, thickening of tubular basement membrane, interstitial accumulation of extracellular matrix (ECM), and fibrosis or scarring^2^. While diabetic tubulointerstitial injury is usually not associated with any specific clinical manifestations or biochemical changes, in particular in the early stage of DKD, it is of crucial significance for the long-term outcome of DKD. As a matter of fact, clinical observations and experimental data suggest that renal tubulointerstitial injury is a histopathologic parameter that correlates best with renal functional impairment for most chronic kidney disease, including DKD^4^. Indeed, while clearly of great importance in DKD, changes in the glomerulus are not the major determinants of renal prognosis in diabetes. Instead, substantial data now support the existence of diabetes-induced renal tubular injury as an early disease event that both predicts and contributes to the development of kidney disease in diabetes^5^.

The pathogenesis of DKD has not been fully understood and is complex and multifactorial, involving genetic, epigenetic, environmental and social-economic determinants^6^. A number of molecular and cellular mechanisms have been implicated in diabetic tubular injury and DKD, including hypoxia, oxidative stress, mitochondria dysfunction, advanced glycation end products, polyol pathways, epithelial-to-mesenchymal transition, metabolic reprogramming and dysregulated expression of growth factors like transforming growth factor (TGF)β1^7^. More recently, diabetes has been shown to be associated with premature senescence of renal cells and accelerated kidney aging^8–11^. However, the underlying mechanism responsible for diabetes-induced premature renal cell senescence remains to be defined and whether this contributes to diabetic kidney injury is elusive.

Serpin E1, also known as plasminogen activator inhibitor 1, has been not only regarded as the key player in hemostasis, fibrinolysis or the normal blood clotting pathway, but also found to exert multiple activities in the regulation of fibrogenesis, cell migration, proliferation and apoptosis^12^. The elevated expression levels of Serpin E1 are related to age and a variety of diseases involved aging process, such as type 2 diabetes, obesity, hypertension and cardiovascular disease^13^. Serpin E1, regulated by TGFβ1, has been reported to play a crucial role in cellular senescence in alveolar type II cells^14^, vascular smooth muscle cells^15^ and glomerular endothelial cells^16^. However, whether TGFβ1 regulation of Serpin E1 contributes to renal tubular senescence in DKD remains unknown. As such, this study utilized the kidney biopsy specimens procured from patients with DKD and employed an in vitro model of diabetic kidney injury to determine the role of Serpin E1 in renal tubular impairments and premature senescence elicited by diabetic insult that involves TGFβ1 signaling.

## Materials and methods

### Human kidney specimens and clinical data

The analysis of kidney specimens and clinical data was approved by the Institutional Review Board of the First Affiliated Hospital of Zhengzhou University in Zhengzhou, China, and conformed to the ethical guidelines of the 1975 Declaration of Helsinki. Human research participants were not specifically recruited for this study. All clinical data and biochemical data were collected by retrospective chart review. All histological data were obtained by examination of archived tissue sections. Excessive or discarded kidney biopsy or nephrectomy specimens had been routinely banked at the Institute of Nephrology of the First Affiliated Hospital of Zhengzhou University. Archived deidentified formalin-fixed paraffin-embedded kidney biopsy tissues from patients with DKD were randomly retrieved for examination. Discarded non-neoplastic nephrectomy specimens without histomorphological lesions were procured from patients who underwent radical nephrectomy due to renal tumor and served as normal controls. Informed consent was obtained from all subjects.

### Bioinformatics analysis

Renal tubulointerstitial transcriptome data of 9 normal kidney tissues from healthy living donors and 10 renal biopsy tissues from patients with DN were generated by the European Renal cDNA Bank (ERCB) nephrotic syndrome study and publicly available from the Nephroseq database (http://www.nephroseq.org). Gene set enrichment analysis (GSEA) was performed based on the renal tubulointerstitial transcriptome data from ERCB subjects that are publicly available from the National Center for Biotechnology Information (NCBI) Gene Expression Omnibus (GEO) database (GEO accession number GSE104954) to identify a possible association of tubulointerstitial TGFβ1 expression with the curated kidney aging-related gene set “RODWELL_AGING_KIDNEY_UP” as previously described^17^. GSEA was performed by employing GSEA v4.1.0 software (http://software.broadinstitute.org/gsea/index.jsp).

### Renal histology assessment and immunohistochemical analysis

Formalin-fixed, paraffin-embedded kidney specimens were prepared as 3-μm-thick sections. For general histology, sections were processed for Masson trichrome staining according to the manufacturer’s instructions (Solarbio Life Science, Beijing, China). The collagen volume fractions of Masson staining were quantified by the ImageJ program (National Institutes of Health, Bethesda, MD). For immunohistochemistry staining, sections were incubated with primary antibody against TGFβ1 (Santa Cruz Biotechnology, TX, USA), p16^INK4A^ (Abcam, MA, USA), Serpin E1 (Santa Cruz Biotechnology), followed by incubation with secondary antibodies and the use of diaminobenzidine substrate kit (Servicebio, Shanghai, China). As negative controls, the primary antibodies were replaced by nonimmune serum from the same species and no nonspecific staining was observed. Computerized morphometry of immunohistochemical staining of TGFβ1, p16^INK4A^ and Serpin E1 was performed as described previously^18^ by using Image-Pro Plus software (Media Cybernetics).

### Cell culture

Immortalized human proximal tubular epithelial cells (HK2) were acquired from the American Type Culture Collection (ATCC, Manassas, VA, USA) and cultured in Dulbecco's modified Eagle's medium (DMEM)/F12 supplemented with 10% fetal bovine serum (FBS). Cells underwent serum starvation for 12 h when reaching 70% confluency. Subsequently, cells were treated for 48 h with a diabetic milieu containing high ambient glucose^19^ (25 mM) with or without TGFβ1 (2 ng/ml; R&D system, MN, USA). Some cultures of cells were additionally treated with the senolytic regimen dasatinib (200 nM, Sigma, MO, USA) plus quercetin (20 μΜ, Sigma) or TM5441 (10 μM; MedChemExpress LLC, NJ, USA). To explore the paracrine effect of differently treated renal tubular cells on other renal tubular epithelial cells, additional cultures of HK2 were treated with the conditioned medium collected from HK2 pre-exposed to different treatments. In brief, HK2 were pre-treated with high osmolality control medium containing 20 mM mannitol or with the diabetic milieu containing high ambient glucose (25 mM) and TGFβ1 for 48 h. Afterwards, cells were changed to fresh normal culture medium. After another 48 h, the conditioned medium was collected and centrifugated at 5000×*g* for 10 min at 4 °C to clear cells and debris. The supernatants were then collected and preincubated with or without the Serpin E1 inhibitor, TM5441 (10 μM) at 37 °C for 1 h to effectively inhibit Serpin E1 before being applied to other cultures of HK2 for 48 h. Finally, cells were processed for detection of senescence-associated β-galactosidase (SA-β-gal) activity, immunofluorescent staining or immunoblot analysis.

### SA-β-gal activity staining

For detection of the SA-β-gal activity, cultured tubular cells were processed by using a commercial kit (Servicebio). SA-β-gal positive cells showed a bright cytoplasmatic blue precipitate. Images were acquired using a phase contrast microscope (EVOS XL, Thermo Fisher Scientific, MA, USA). The results were presented as the absolute count of SA-β-gal positive cells expressed as a percentage of the total number of cells per high-power field.

### Immunofluorescent staining

Cultured renal tubular cells were fixed with 4% paraformaldehyde and incubated overnight with primary antibody against fibronectin (Santa Cruz Biotechnology), followed by Alexa Flour 594 conjugated-secondary antibody staining (Invitrogen, CA, USA). Finally, cells were mounted with mounting media containing 4,6-diamidino-2-phenylindole (Vector Laboratories, CA, USA) and visualized using the Nikon upright microscope (Nikon Instruments, Shanghai, China).

### Western immunoblot analysis

Cells were lysed in radioimmunoprecipitation assay buffer supplemented with the protease inhibitor and phenylmethylsulfonyl fluoride. Cell lysates were subjected to western immunoblot analysis as described before^20^. The blots were incubated with specific antibodies against E-cadherin (Cell Signaling Technology), fibronectin (Santa Cruz Biotechnology), Serpin E1(Santa Cruz Biotechnology), p53 (Santa Cruz Biotechnology), p16^INK4A^ (Abcam), and GAPDH (Santa Cruz Biotechnology). The integrated pixel density of immunoblot bands was determined using the ImageJ program.

### Statistical analysis

Data were presented as mean ± SD or median (interquartile range). All in vitro studies were repeated at least 3 times. The normality of all data was tested with the Shapiro–Wilk normality test. Comparisons among multiple groups were performed using one-way ANOVA followed by Tukey’s test. Data from two groups were compared by 2-tailed, unpaired Student’s t test. Linear regression analysis was applied to test possible relationships between 2 parameters. *P* < 0.05 was considered statistically significant.

## Results

### Increased expression of TGFβ1 in renal tubulointerstitia is evident in diabetic nephropathy and associated with kidney aging

**Figure 1:**
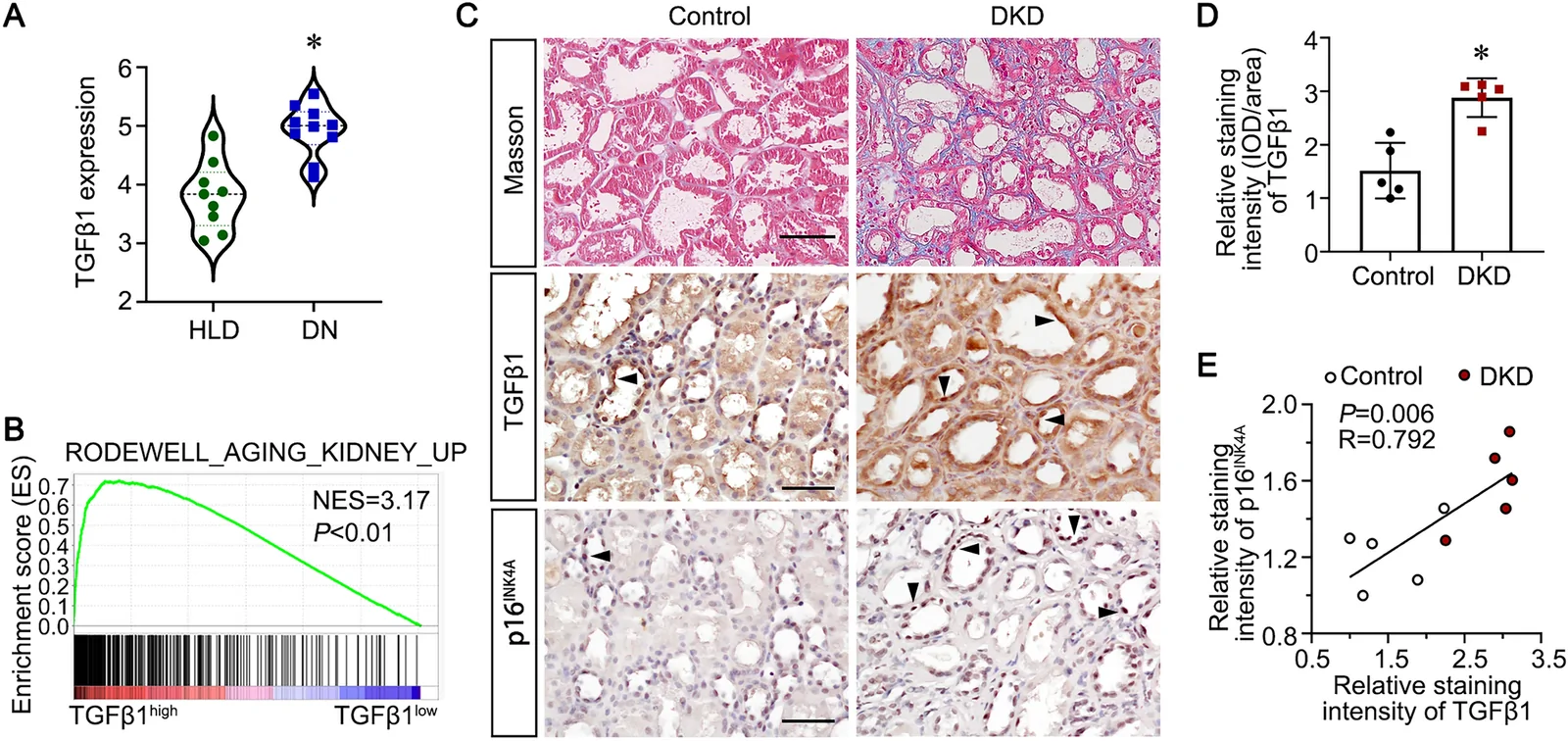
TGFβ1 is overexpressed in tubulointerstitia and associated with senescence in diabetic nephropathy. (**A**) Post hoc analysis of TGFβ1 expression levels in the tubulointerstitium of healthy living donors (HLD) or diabetic nephropathy (DN). The transcriptome data were derived from www.Nephroseq.org based on the ERCB nephrotic syndrome datasets and expressed as Log2 median-centered intensity. *P < 0.05 versus healthy living donor group (n = 9–10). (**B**) Gene set enrichment analysis (GSEA) demonstrated that the predefined gene sets “RODEWELL_KIDNEY_AGING_UP” exhibited significant enrichment in high-expression of TGFβ1 *versus* low-expression of TGFβ1 in renal tubulointerstitial specimens procured from HLD and patients with DN based on data derived from GSE104954 dataset. Normalized enrichment score (NES) and nominal *P* value are shown. (**C**) Kidney specimens derived from patients with diabetic kidney disease (DKD) were subjected to Masson trichrome staining or immunohistochemistry staining for TGFβ1 or p16^INK4A^. Non-neoplastic nephrectomy specimens procured from patients with renal tumors served as controls. Scale bar = 50 μm. (**D**) The immunohistochemical staining intensity of TGFβ1 was estimated by computerized morphometric analysis. IOD, integrated optical density. *P < 0.05 versus control group (n = 5). (**E**) Linear regression analyses of the relative staining intensity of TGFβ1 and p16^INK4A^. *P* value and Spearman’s correlation coefficient (*R*) are shown. (n = 5 per group).

As a growth factor centrally involved in chronic fibrogenesis in most organ systems, TGFβ1 plays a key role in the development and progression of chronic kidney diseases, including DKD^21^. Recently, TGFβ1 has been shown to possess a pro-senescent activity and to be a quintessential senescence-associated secretory phenotype (SASP). However, it is unknown whether TGFβ1 mediates diabetes-induced premature senescence of renal parenchymal cells. To address this issue, a post hoc analysis of the renal tubulointerstitial transcriptome was conducted based on the publicly available Nephroseq data derived from the “ERCB Nephrotic Syndrom Tublnt” transcriptomic datasets of kidney biopsy tissues procured from patients with DN and healthy living donors. As shown in Fig. 1A, renal tubulointerstitial expression of TGFβ1 was significantly higher in DN group than that in normal controls. In addition, as demonstrated by GSEA, the curated kidney-aging-related gene set “RODWELL_AGING_KIDNEY_UP” exhibited significant enrichment in high expression of TGFβ1 as compared with low expression of TGFβ1 in renal tubulointerstitial specimens procured from patients with DN or healthy living donors based on the ERCB study (Fig. 1B). Collectively, these findings suggest that TGFβ1 is overexpressed in renal tubulointerstitia in DN as opposed to healthy living controls, associated with accelerated kidney aging in diabetes.

### Amplified expression of TGFβ1 in renal tubules correlates with renal tubular premature senescence and degenerative changes in DKD

To verify the bioinformatics data, archived kidney biopsy specimens collected from patients with DKD were retrieved and examined. As normal controls, another series of kidney biopsies derived from non-neoplastic nephrectomy specimens were examined simultaneously. The two groups of patients were matched for age, sex and comorbidities, as revealed in Supplementary Table 1. Sections of kidney biopsy specimens were processed for Masson trichrome staining or peroxidase immunohistochemistry staining for TGFβ1 and p16^INK4A^. Shown in Fig. 1C and D, TGFβ1 was more intensely and diffusely expressed in kidneys in the DKD group as compared with that in the control group, with predominant expression located in renal tubules. This was associated with increased expression of the senescent marker p16^INK4A^ in renal tubules in the same sections in the DKD group. Computerized morphometric analysis indicated that the relative staining intensity of TGFβ1 positively correlated with that of p16^INK4A^ (Fig. 1E). In parallel, Masson trichrome staining demonstrated that the same kidney section with intense TGFβ1 and p16^INK4A^ staining exhibited prominent chronic degenerative changes, including renal tubular atrophy marked by flattened and simplified tubular epithelia, thickening of tubular basement membrane, and accumulation of ECM (Fig. 1C).

### Diabetic insult induces premature cellular senescence and degenerative changes in renal tubular epithelial cells

**Figure 2:**
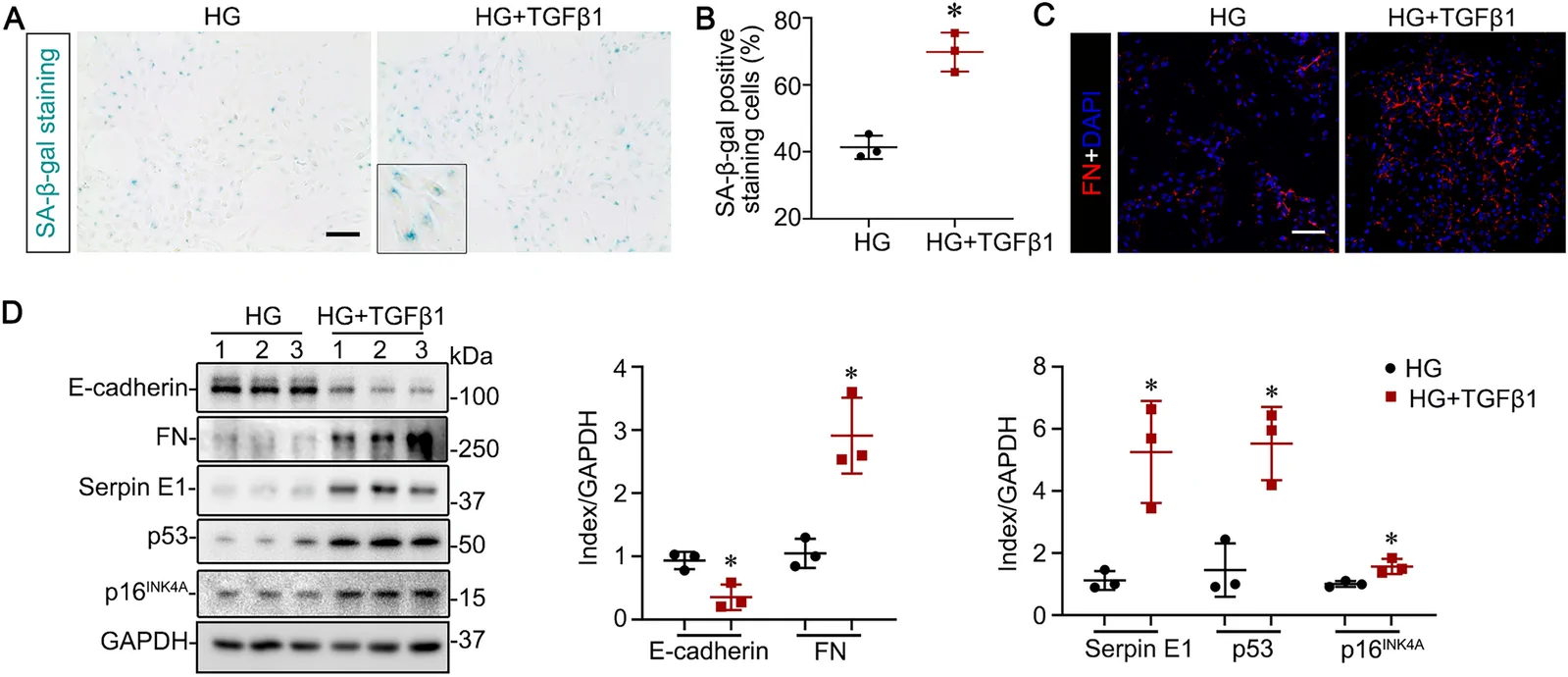
TGFβ1 elicited tubular senescence and senescence-associated secretory phenotypes (SASPs) in diabetic milieu in vitro. Cultured tubular cells were treated with high ambient glucose (HG) in the presence or absence of TGFβ1. (**A**) Cells were processed for SA-β-gal activity staining. Representative micrographs were shown. Scale bar = 50 μm (**B**) Quantification of the SA-β-gal positive cells as percentages of the total number of cells per microscopic field. *P < 0.05 versus HG group (n = 3). (**C**) Cells were processed with immunofluorescent staining for fibronectin (FN) followed by counterstaining with DAPI. Scale bar = 100 μm. (**D**) Cell lysates were processed for immunoblot analysis for indicated proteins, followed by densitometry analysis, presented as relative levels normalized to GAPDH based on immunoblot analysis. *P < 0.05 versus the same protein expression in HG group (n = 3).

Diabetes may accelerate aging-like changes in the kidney that are characterized by premature cellular senescence. To test if TGFβ1 has a direct pro-senescent effect on renal tubular cells in DKD, an in vitro model of DKD was adopted by treating cultured renal tubular epithelial cells with a diabetic milieu simulated by high ambient glucose and TGFβ1 treatments. High ambient glucose alone slightly triggered cellular senescence as probed by staining for the acidic SA-β-gal activity (Supplemental Fig. 1), one of the most reliable biomarkers of cellular senescence. In contrast, the combination of high ambient glucose with TGFβ1 injury strikingly increased the staining for SA-β-gal activity as well as the number of cells positive for SA-β-gal activity staining (Fig. 2A,B), suggestive of a pro-senescent effect. This was associated with increased expression of p16^INK4A^ and p53, key signaling mediators of the cellular senescence pathways^22^, as determined by immunoblot analysis. In parallel, the expression of Serpin E1, a typical SASP factor, was induced, coinciding with increased expression of the ECM component fibronectin and reduced expression of the epithelial marker E-cadherin, as shown by immunofluorescent staining (Fig. 2C) or immunoblot analysis (Fig. 2D). These findings are all indicative of degenerative changes of tubular epithelial cells upon diabetic insult.

### Senolytic treatment abrogates the senescence response and attenuates degenerative changes in renal tubular epithelial cells exposed to the diabetic milieu

**Figure 3:**
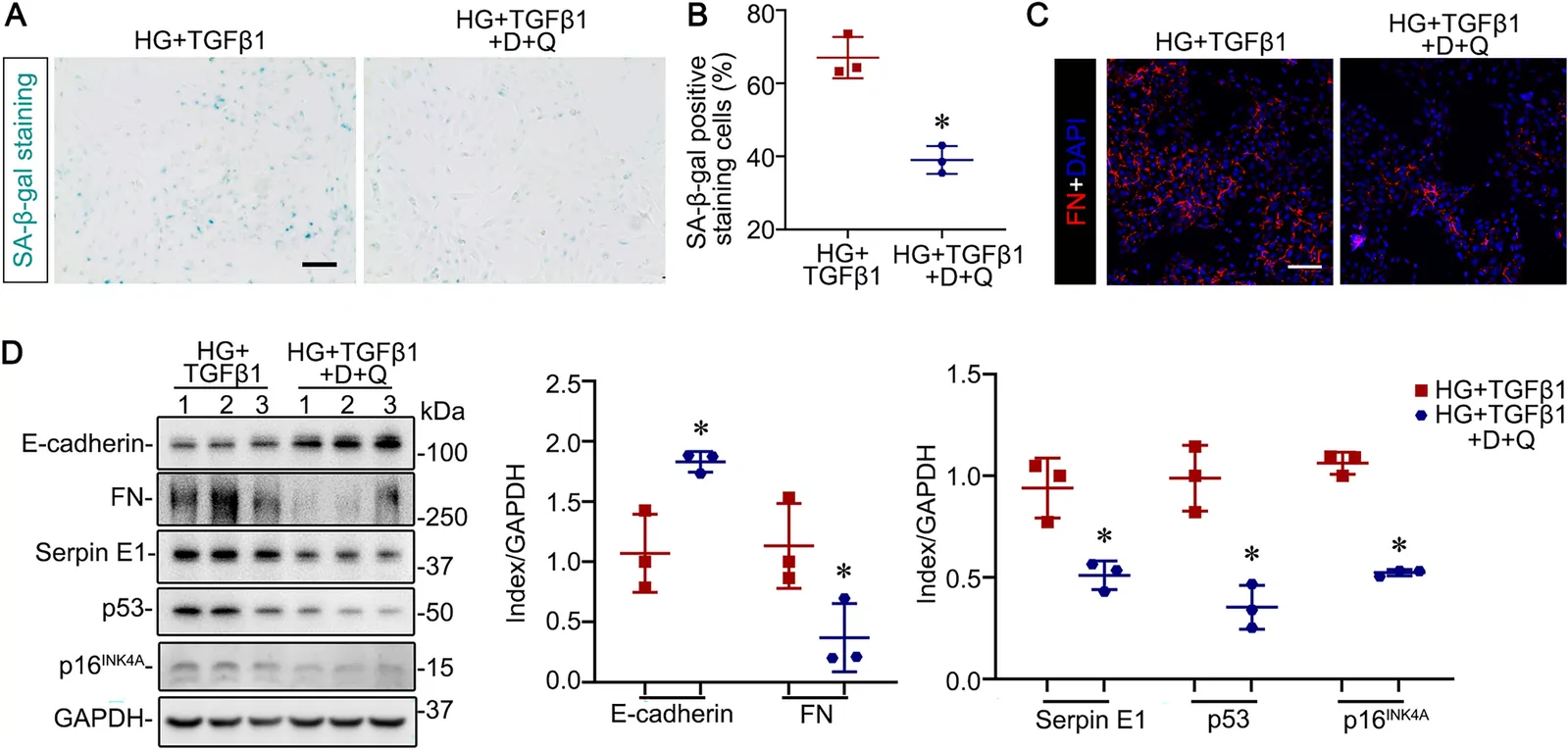
TGFβ1 induced tubular senescence and degeneration was ameliorated by senolytics. (**A**) Cultured tubular cells were exposed to HG (25 mM) + TGFβ1 (2 ng/ml) in the presence or absence of dasatinib (200 nM) and quercetin (20 μM) (D + Q). (A) Cells were processed for SA-β-gal activity staining. Scale bar = 50 μm. (**B**) Quantification of the SA-β-gal positive cells as percentages of the total number of cells per microscopic field. *P < 0.05 versus HG + TGFβ1 group (n = 3). (**C**) Cells were processed for immunofluorescent staining for fibronectin (FN) followed by counterstaining with DAPI. Scale bar = 100 μm. (**D**) Cell lysates were processed for immunoblot analysis for indicated proteins, followed by densitometry analysis, presented as relative levels normalized to GAPDH based on immunoblot analysis. *P < 0.05 versus the same protein expression in HG + TGFβ1 group (n = 3).

Recent evidence suggests that premature senescence is likely an important mechanism for the pathogenesis of a number of diseases including DKD^23^. To determine if TGFβ1-elicited senescence plays a key role in renal tubular impairment and degenerative changes in DKD, additional cells were treated with the TGFβ1-containing diabetic milieu in the presence or absence of a well-established senolytic regimen consisting of dasatinib and quercetin. As shown in Fig. 3A,B, dasatinib and quercetin treatment substantially abrogated the staining for SA-β-gal activity in renal tubular epithelial cells exposed to the TGFβ1-containing diabetic milieu, consistent with a powerful anti-senescent activity of dasatinib and quercetin. This was associated with reduced induction of p16^INK4A^, p53, Serpin E1 and fibronectin (Fig. 3C,D). In contrast, the expression of E-cadherin was significantly preserved, denoting a protective effect on renal tubular cells that mitigates cellular degeneration (Fig. 3D). Collectively, these findings suggest that the pro-senescent effects of TGFβ1 contribute, at least in part, to renal tubular impairments upon diabetic insult.

### Inhibiting Serpin E1 improves renal tubular cell senescence and impairments upon diabetic insult

**Figure 4:**
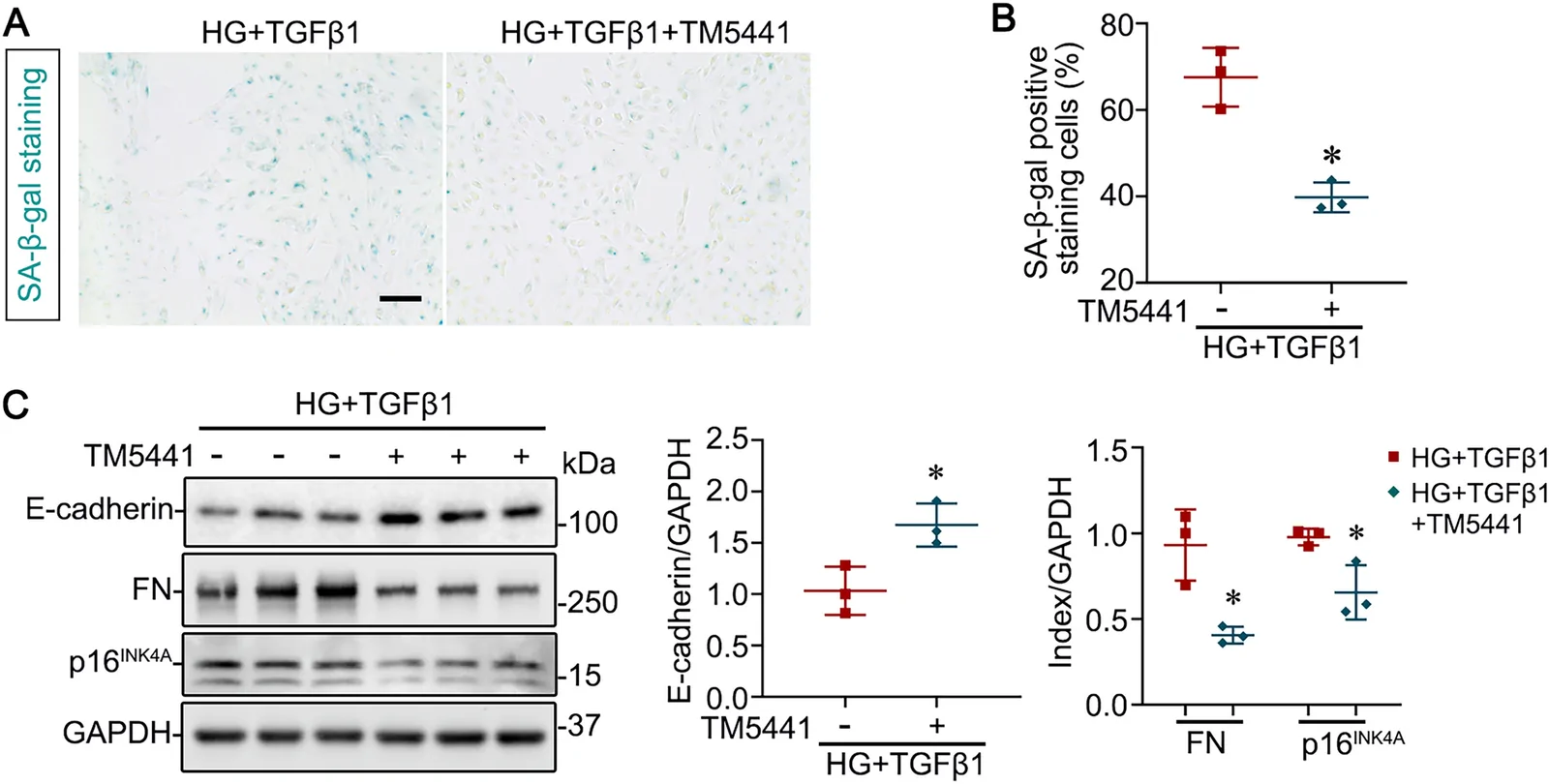
Inhibition of Serpin E1 mitigated tubular cell senescence elicited by TGFβ1 in diabetic milieu. Cells were treated with high glucose (HG, 25 mM) and TGFβ1 (2 ng/ml) in the presence or absence of TM5441 (10 μM) for 48 h. (**A**) Cells were processed for SA-β-gal activity staining. Scale bar = 50 μm. (**B**) Quantification of the SA-β-gal positive cells as percentages of the total number of cells per microscopic field. *P < 0.05 versus HG + TGFβ1 group (n = 3). (**C**) Cell lysates were processed for immunoblot analysis for fibronectin (FN), p16^INK4A^ and GAPDH, followed by densitometry analysis, presented as relative levels normalized to GAPDH based on immunoblot analysis. *P < 0.05 versus the same protein expression in HG + TGFβ1 group (n = 3).

Among numerous downstream targets of TGFβ1, Serpin E1 is regarded as a SASP factor with potent pro-senescence effects in a number of cell types^24^, such as alveolar type II cells^25^, keratinocytes^26^ and vascular cells^27^. To determine whether Serpin E1 is involved in TGFβ1-elicited tubular cell senescence in DKD. Tubular epithelial cells were treated with or without TM5441, a highly selective small molecule inhibitor of Serpin E1, before exposure to high ambient glucose and TGFβ1. As shown in Fig. 4A and B, TM5441 treatment significantly diminished the intensity of staining for SA-β-gal activity and the number of cells positive for SA-β-gal in renal tubular epithelial cells exposed to the TGFβ1-containing diabetic milieu, reminiscent of the effect of senolytic drugs dasatinib and quercetin (Fig. 3). In concert, the expression of p16^INK4A^ was markedly decreased as shown by immunoblot analysis followed by densitometry (Fig. 4C). In addition, the tubular degenerative changes were also mitigated by TM5441, as shown by preserved expression of E-cadherin and decreased expression of fibronectin (Fig. 4C). These findings suggest the mediating role of Serpin E1 in TGFβ1-induced tubular cell senescence upon diabetic insult.

### Serpin E1 is required for the paracrine senescence of renal tubular cells upon diabetic insult

**Figure 5:**
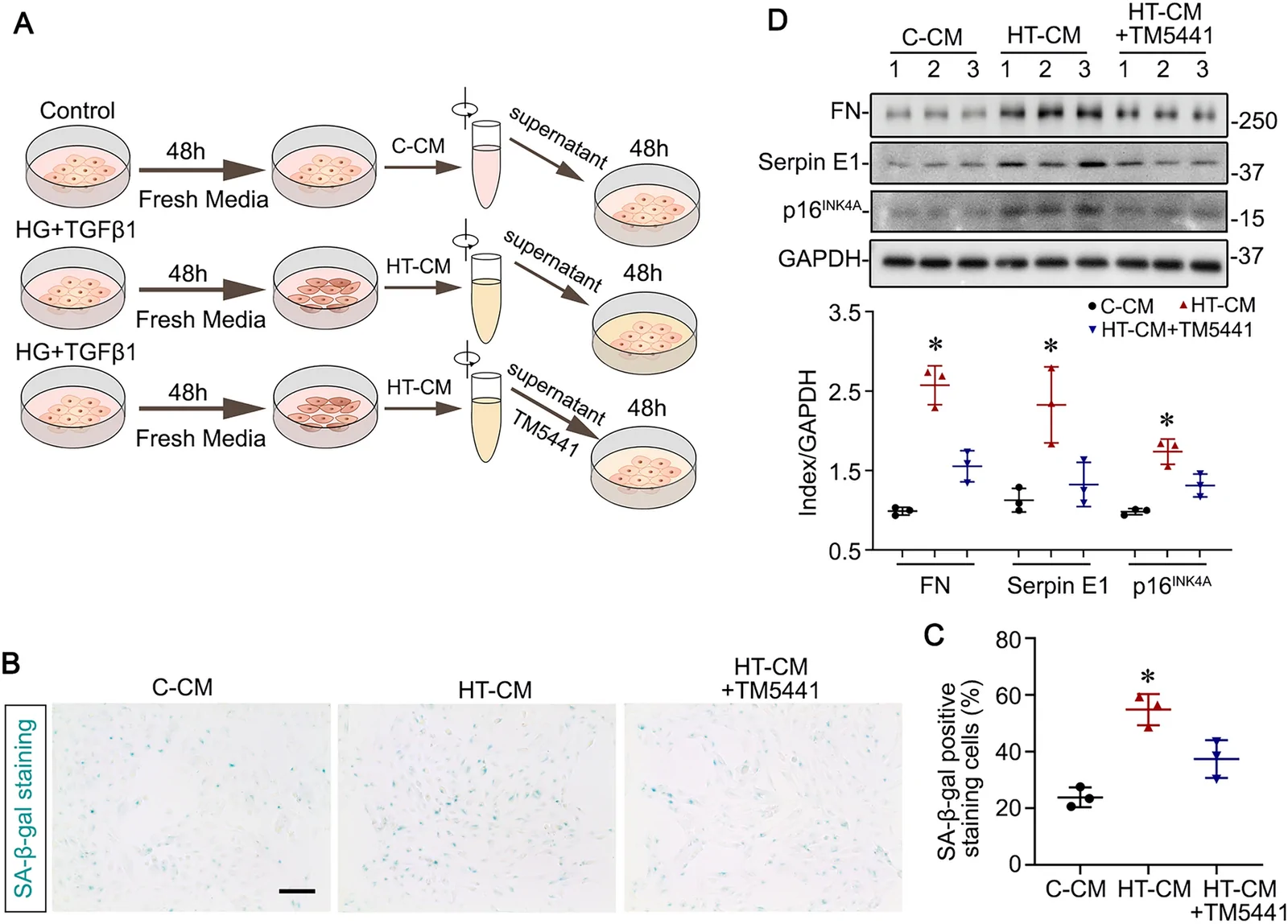
Targeting secretory Serpin E1 attenuated TGFβ1-induced paracrine senescence in cultured tubular cells. (**A**) Schematic diagram depicts the study design. Cells were treated with high osmolality control medium containing 20 mM mannitol or the diabetic milieu containing HG (25 mM) + TGFβ1 (2 ng/ml) for 48 h. Thereafter, cells were changed to the fresh normal culture medium. After 48 h, the conditioned medium (CM) was collected and centrifuged. The supernatants of CM were preincubated with or without TM5441 before being applied to separate cultures of cells for 48 h. (**B**) Cells were processed for SA-β-gal activity staining. Scale bar = 50 μm. (**C**) Quantification of the SA-β-gal positive cells as percentages of the total number of cells per microscopic field. *P < 0.05 versus all other groups (n = 3). (**D**) Cell lysates were processed for immunoblot analysis for indicated proteins, followed by densitometry analysis, presented as relative levels normalized to GAPDH based on immunoblot analysis. *P < 0.05 versus the same protein expression in other groups (n = 3). C-CM, conditioned medium from control medium containing 20 mM mannitol treated cells; HT-CM, conditioned medium from HG + TGFβ1 treated cells.

As a typical SASP factor, Serpin E1 may convey the intercellular communication between the senescent cells and other cells^28,29^, thereby expanding the effects of the original injury. This prompted us to test if Serpin E1 plays a role in the influence of senescent renal tubular cells on other renal tubular cells. Renal tubular epithelial cells were exposed to the TGFβ1-containing diabetic milieu as elaborated above for 48 h, and then were changed to normal medium. After 48 h, the conditioned medium was collected and processed to treat additional cultures of normal renal tubular cells for 48 h (Fig. 5A). The conditioned medium derived from the TGFβ1-containing diabetic milieu-injured cells, as opposed to that from control cells treated with high osmotic mannitol, evidently induced cellular senescence in cultures of normal renal tubular cells, as shown by the staining for SA-β-gal activity (Fig. 5B,C). This was associated with increased expression of p16^INK4A^ and fibronectin, indicative of paracrine senescence and degenerative changes (Fig. 5D). These detrimental effects were largely abolished by the addition of the Serpin E1 inhibitor TM5441 to the conditioned medium (Fig. 5D), suggesting that Serpin E1 is essential for the paracrine senescence of renal tubular cells upon diabetic insult.

### Renal tubular expression of Serpin E1 is increased in DKD and correlates with renal tubular premature senescence and degenerative changes

**Figure 6:**
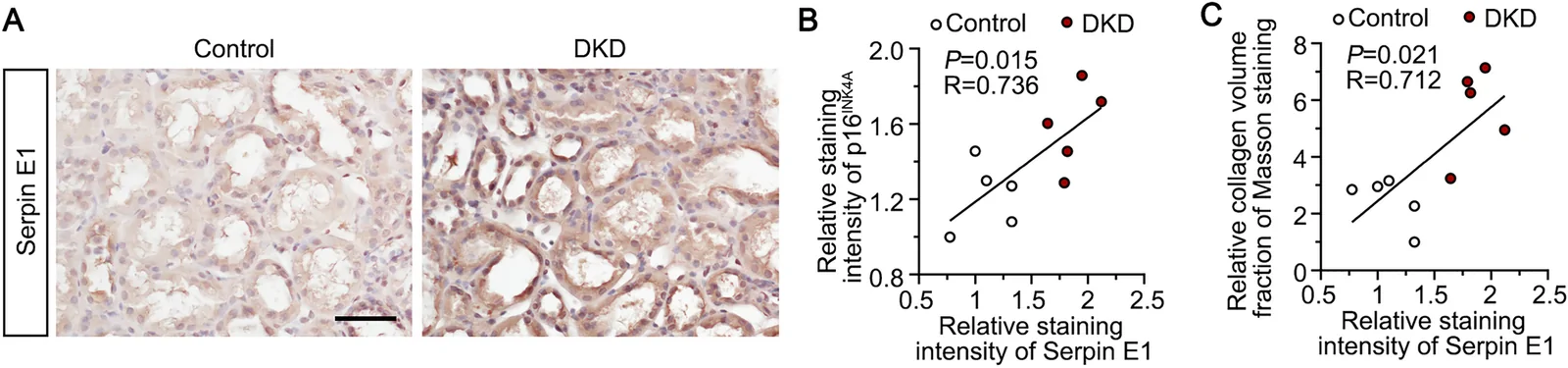
Increased expression of Serpin E1 is evident in renal tubules in diabetic kidney disease, correlating with renal tubular senescence and degenerative changes. (**A**) Kidney specimens derived from patients with diabetic kidney disease (DKD) were subjected to peroxidase immunohistochemistry staining for Serpin E1. Non-neoplastic nephrectomy specimens procured from patients with renal tumors served as controls. Scale bar = 50 μm. (**B**,**C**) Linear regression analyses of the staining intensity of Serpin E1 and (**B**) that of p16^INK4A^ (Fig. 1C) or (**C**) collagen volume fraction of Masson trichrome staining (Fig. 1C). *P* value and Spearman’s correlation coefficient (*R*) are shown. (n = 5 per group).

To ascertain if Serpin E1 expression is altered in diabetic kidneys and if this is involved in diabetes-accelerated kidney aging in DKD, kidney specimens derived from patients with DKD or normal control kidney tissues were subjected to peroxidase immunohistochemistry staining for Serpin E1. As shown in Fig. 6A, Serpin E1 was faintly detected in normal kidney tissues with very weak staining located to renal tubules. In contrast in kidneys with DKD, intense staining of Serpin E1 was noted in renal tubules and interstitia with the strongest expression located to atrophic renal tubules marked by simplified and flattened renal tubular epithelia surrounded by a wrinkled and thickened tubular basement membrane. Moreover, computerized morphometric analysis of immunohistochemistry staining of Serpin E1 (Fig. 6A) and p16^INK4A^ (Fig. 1C) as well as Masson trichrome staining (Fig. 1C) revealed that the relative staining intensity of Serpin E1 positively correlated with that of p16^INK4A^ (Fig. 6B) or collagen volume fraction of Masson staining (Fig. 6C) in control and diabetic kidneys, suggesting that increased renal tubular expression of Serpin E1 is associated with renal tubular premature senescence and degenerative changes in DKD.

## Discussion

The molecular mechanisms driving the development and progression of DKD are not fully understood. Recent evidence suggests that diabetes is associated with accelerated or premature aging in various tissues, including the kidney, and this effect may play a key role in the pathogenesis of diabetic complications like DKD^10^. However, how premature renal aging is elicited in DKD is obscure. To the best of our knowledge, this study is the first one to demonstrate that Serpin E1 is a critical mediator of diabetic insult in the induction of renal tubular degeneration and premature senescence in DKD.

A myriad of factors has been involved in the pathogenesis of DKD and thus may be implicated in diabetes-accelerated kidney aging. Among these, high ambient glucose has been shown to cause cellular senescence in cultured renal cells^30,31^. Indeed, glycemic control may have some benefits in slowing the progression of DKD. However, clinical evidence suggests that not every diabetic patient develops DKD despite poor glycemic control, arguing that high glucose alone is unlikely a major determinant of diabetes-accelerated kidney aging and DKD^32^. Conversely, even with strict glycemic control, some patients still develop DKD, suggesting that some unknown mediators cause kidney injury.

Besides hyperglycemia, growth factors like TGFβ1 have been implicated in DKD as a fundamental profibrogenic cytokine^33^. In addition to being a key factor that drives fibrosis, TGFβ1 has been implicated in cell senescence and aging-related pathology^34^. For instance, Alzheimer’s disease is associated with an increase of TGFβ in the brain and cerebrospinal fluid^35^. Muscle atrophy is also associated with muscle levels of TGFβ1^36^. In concert, TGFβ1 overexpression has also been associated with DKD, as reported previously^21,37^ and also shown by the present study. However, it is uncertain if TGFβ1 elicits senescence of renal parenchymal cells and if this effect contributes to DKD. Our data indicate that TGFβ1 treatment in the presence of high ambient glucose evidently elicited renal tubular cell senescence, in parallel with signs of degenerative changes, including tubular cell dedifferentiation, marked by loss of E-cadherin expression, and profibrogenic changes, shown by ECM accumulation. Importantly, co-treatment with the typical senolytic regimen of dasatinib plus quercetin effectively eliminated TGFβ1-elicited senescence, resulting in the abrogation of signs of degenerative changes. These data suggest that TGFβ1-elicited senescence plays a key role in diabetes-associated renal tubular injury.

**Figure 7:**
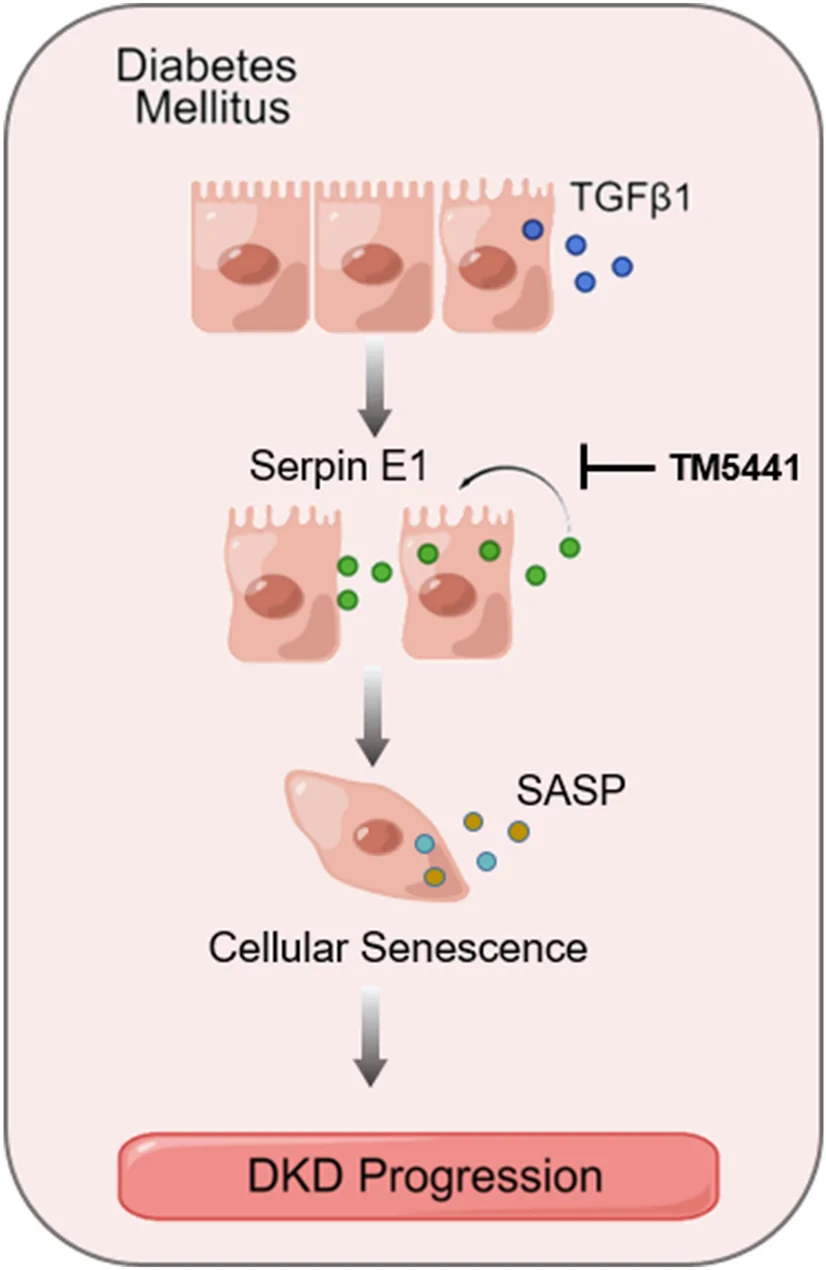
Schematic depicts the critical role of TGFβ1/Serpin E1 in promoting diabetes-accelerated kidney aging and injury via driving renal tubular premature senescence and degeneration. In diabetes mellitus, elevated renal expression of TGFβ1 promotes Serpin E1 secretion in tubular epithelial cells, which, in turn, act on cells in an autocrine or paracrine manner. Subsequently, Serpin E1 elicits renal tubular senescence, characterized by staining for senescence-associated β-galactosidase activity, increased p16^INK4A^ expression and senescence-associated secretory phenotypes. These effects work synergistically to contribute to tubular cell degeneration and impairments, and ultimately lead to diabetic kidney disease progression. This pathogenic pathway could be intercepted by TM5441, a highly selective small molecule inhibitor of Serpin E1.

It is not fully understood how TGFβ1 induces a senescence response in renal tubular cells upon diabetic insult. Of note, TGFβ1 is a potent inducer of Serpin E1 that in turn mediates a number of biological effects of TGFβ1^38^. In patients with DKD, it has been reported that the overexpression of Serpin E1 was evident in kidney tissues^39^ and plasma^40^, and positively correlated with the severity of albuminuria^40^. In animal models of DKD, inhibition or deletion of Serpin E1 attenuated diabetes-associated albuminuria and glomerular injury^41,42^. The detrimental effects of Serpin E1 in DKD have been assumed to be attributable to its inhibitory activity on extracellular matrix degeneration^42,43^. Nevertheless, emerging studies suggested that Serpin E1 is also able to promote cellular senescence^25,44,45^, and thereby contributes to lung fibrosis^14^, hypertension-induced arteriosclerosis^27^, as well as pulmonary aging^46^. In agreement, in our study, the Serpin E1 inhibitor markedly abolished senescence and degenerative changes in renal tubular cells exposed to the diabetic milieu that contains TGFβ1, implying the essential role of Serpin E1 in mediating TGFβ1-elicited senescence in DKD. In addition, the conditioned medium collected from renal tubular cells treated with the diabetic milieu was able to promote senescence and induce signs of degenerative changes in separate cultures of healthy renal tubular cells. This effect was abolished by the Serpin E1 inhibitor, suggesting the critical role of Serpin E1 in mediating the paracrine senescence of renal tubular cells in DKD. Given the predominant composition of renal tubular cells in the renal parenchyma, it is conceivable that TGFβ1/Serpin E1-elicited senescence may be expanded slowly but persistently throughout the nephron and the whole kidney via this paracrine signaling, thus representing a final common pathway for diabetes-associated kidney injury (Fig. 7).

Our study is not without limitations. The model system employed in this study is an oversimplified one of diabetic kidney injury and does not fully recapitulate the pathogenesis of DKD, in particular DKD secondary to type 2 diabetes mellitus, which often coincides with multiple comorbidities such as hypertension, dyslipidemia and hyperuricemia. In addition to high ambient glucose and TGFβ1 used in the present study, other mediators are also involved in DKD and may contribute to diabetes-associated cellular senescence in the kidney. Among these, vasoactive mediators like angiotensin II have been shown to induce senescence of renal endothelial cells^47^. In addition, microinflammation associated with hyperuricemia or uric acid per se may exert a pro-senescent and aging effect^48^. Moreover, chronic hyperinsulinemia has recently been associated with cell cycle exit in adipocytes both in vitro and in vivo in humans, leading to a premature senescent transcriptomic and secretory profile^49^. Whether angiotensin II or exposure to high ambient insulin or uric acid promotes senescence in renal parenchymal cells like renal tubular cells is unknown and merits further investigations in the future. Our study by no means rules out the contribution of other pathogenic mediators or pathways to diabetes-associated premature senescence and degenerative changes of renal tubules.

In summary, increased TGFβ1 expression was associated with premature senescence and degeneration of renal tubules in patients with DKD. In cultured renal tubular epithelial cells exposed to the diabetic milieu containing TGFβ1, Serpin E1 was a critical mediator of renal tubular cell senescence and degeneration, and was essential for inducing paracrine senescence (Fig. 7). Our study demonstrated that Serpin E1 may be harnessed as a novel actionable therapeutic target for treating DKD via targeting diabetes-associated premature senescence and degeneration.

## Supplementary Information

Supplementary Information 1.

Supplementary Information 2.

## Data Availability

The underlying data are available from the corresponding author upon reasonable request.
