# Correction: Serpin E1 mediates the induction of renal tubular degeneration and premature senescence upon diabetic insult

**DOI:** 10.1038/s41598-026-70468-8

**Published:** 2026-09-15

**Authors:** Bo Han Chen, Xiao Qing Lu, Xian Hui Liang, Pei Wang

**Affiliations:** 1 Blood Purification Center, The First Affiliated Hospital of Zhengzhou University, Zhengzhou, China; 2 Research Institute of Nephrology, Zhengzhou University, Zhengzhou, China; 3 Blood Purification Center, Department of Nephrology, The First Affiliated Hospital of Zhengzhou University, 1 East Jianshe Road, Zhengzhou, 450052 Henan China

Correction to: *Scientific Reports* https://doi.org/10.1038/s41598-023-43411-4, published online 27 September 2023

The original version of this Article contains errors in panel D of Figure 2.

As a result of an oversight during figure assembly, an incorrect blot image is presented for the “GAPDH” panel. The associated densitometric analysis was reperformed by the authors using the correct blot image, and the results remained consistent with the original findings.

The correct Figure 2 and accompanying legend appear below.

Additionally, in the Supplementary file that accompanies the original Article, an incorrect blot image is presented for “GAPDH”. The corrected Supplementary file is provided below.

**Fig. 2:**
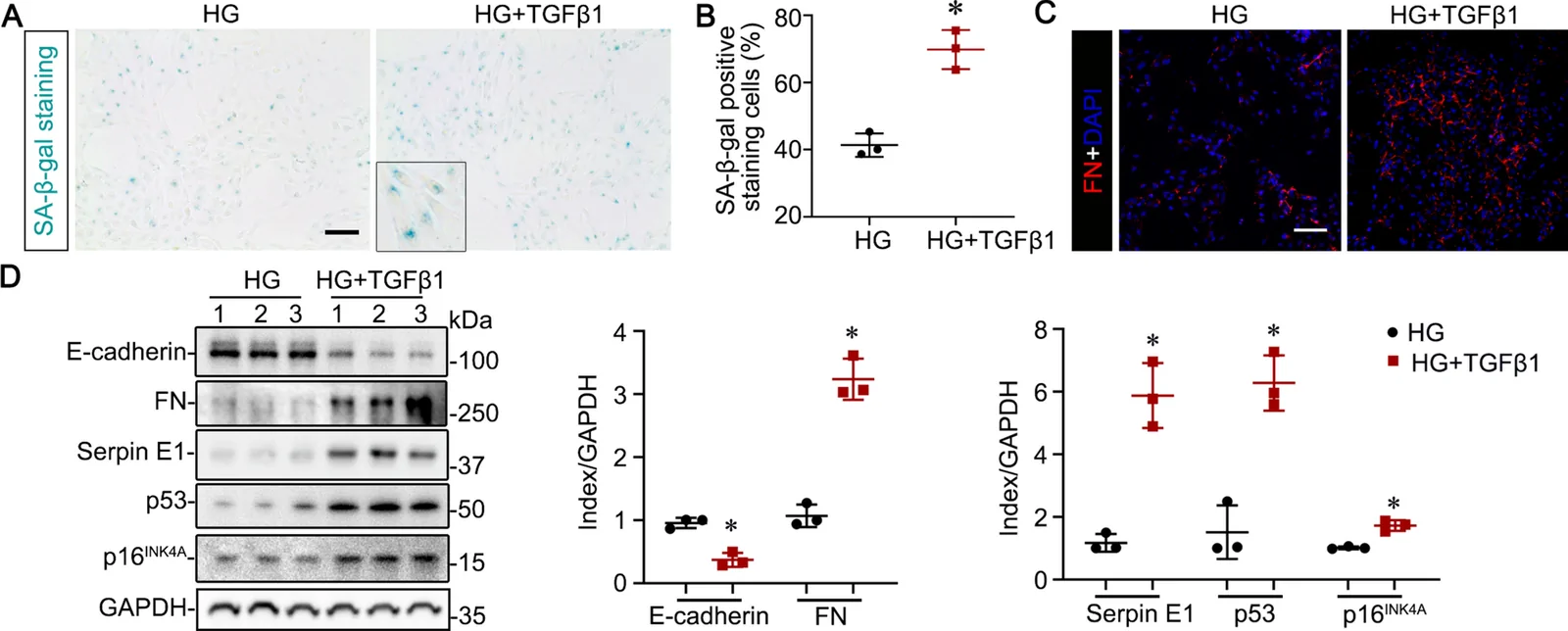
TGFβ1 elicited tubular senescence and senescence-associated secretory phenotypes (SASPs) in diabetic milieu in vitro. Cultured tubular cells were treated with high ambient glucose (HG) in the presence or absence of TGFβ1. (**A**) Cells were processed for SA-β-gal activity staining. Representative micrographs were shown. Scale bar = 50 μm (**B**) Quantification of the SA-β-gal positive cells as percentages of the total number of cells per microscopic field. *P < 0.05 versus HG group (n = 3). (**C**) Cells were processed with immunofluorescent staining for fibronectin (FN) followed by counterstaining with DAPI. Scale bar = 100 μm. (**D**) Cell lysates were processed for immunoblot analysis for indicated proteins, followed by densitometry analysis, presented as relative levels normalized to GAPDH based on immunoblot analysis. *P < 0.05 versus the same protein expression in HG group (n = 3).

## Supplementary Information

Supplementary Information.

