# Supplementary material for: Serpin E1 mediates the induction of renal tubular degeneration and premature senescence upon diabetic insult
Source: Sci Rep. 2023 Sep 27;13:16210. doi: 10.1038/s41598-023-43411-4 (PMC10533493; doi:10.1038/s41598-023-43411-4)
Supplement: Supplementary file 1 — Supplementary Information 1. [file 41598_2023_43411_MOESM1_ESM.pdf]

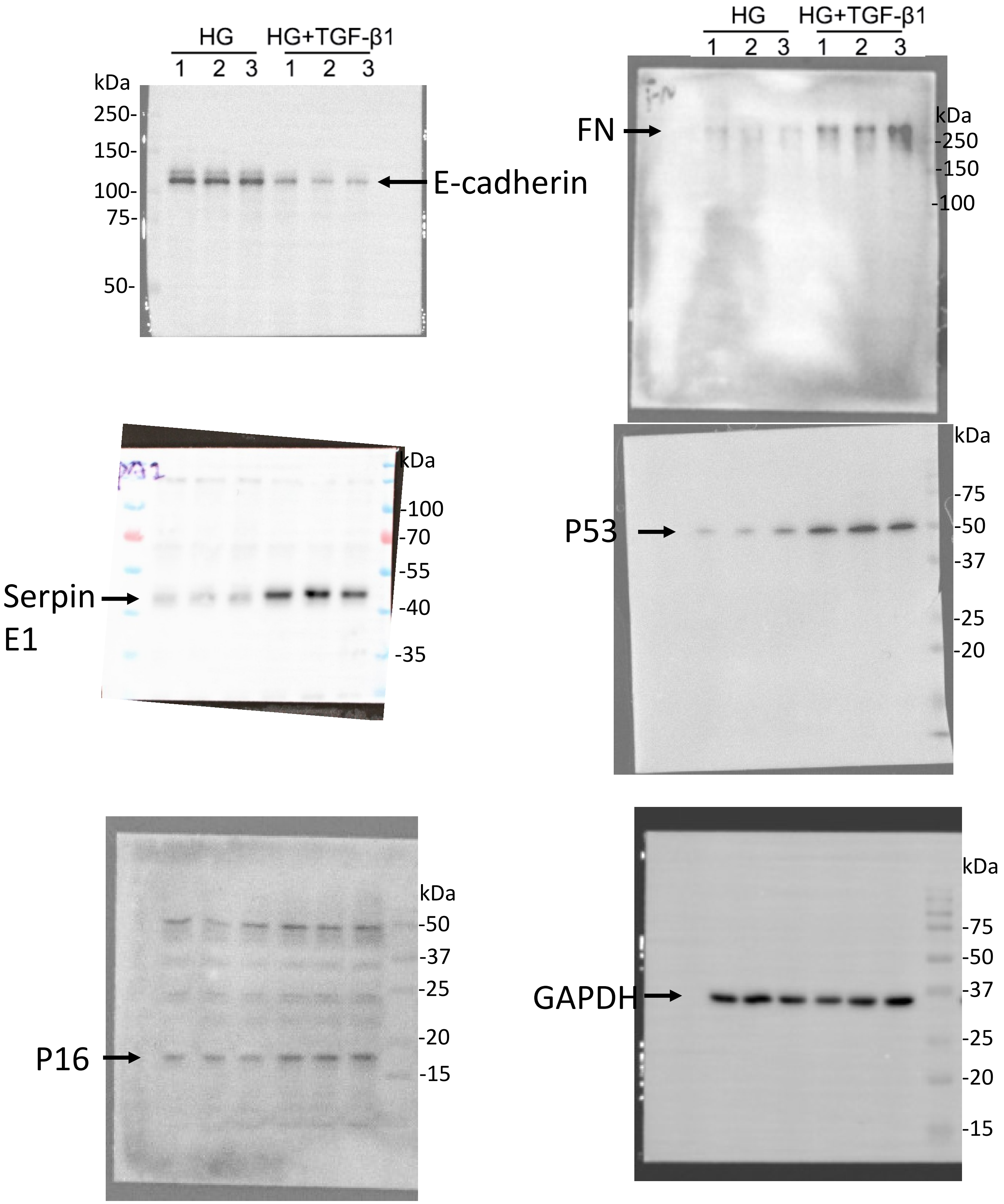

Source Data Figure 1: Uncropped blots used for the construction of composite of Figure 2D.

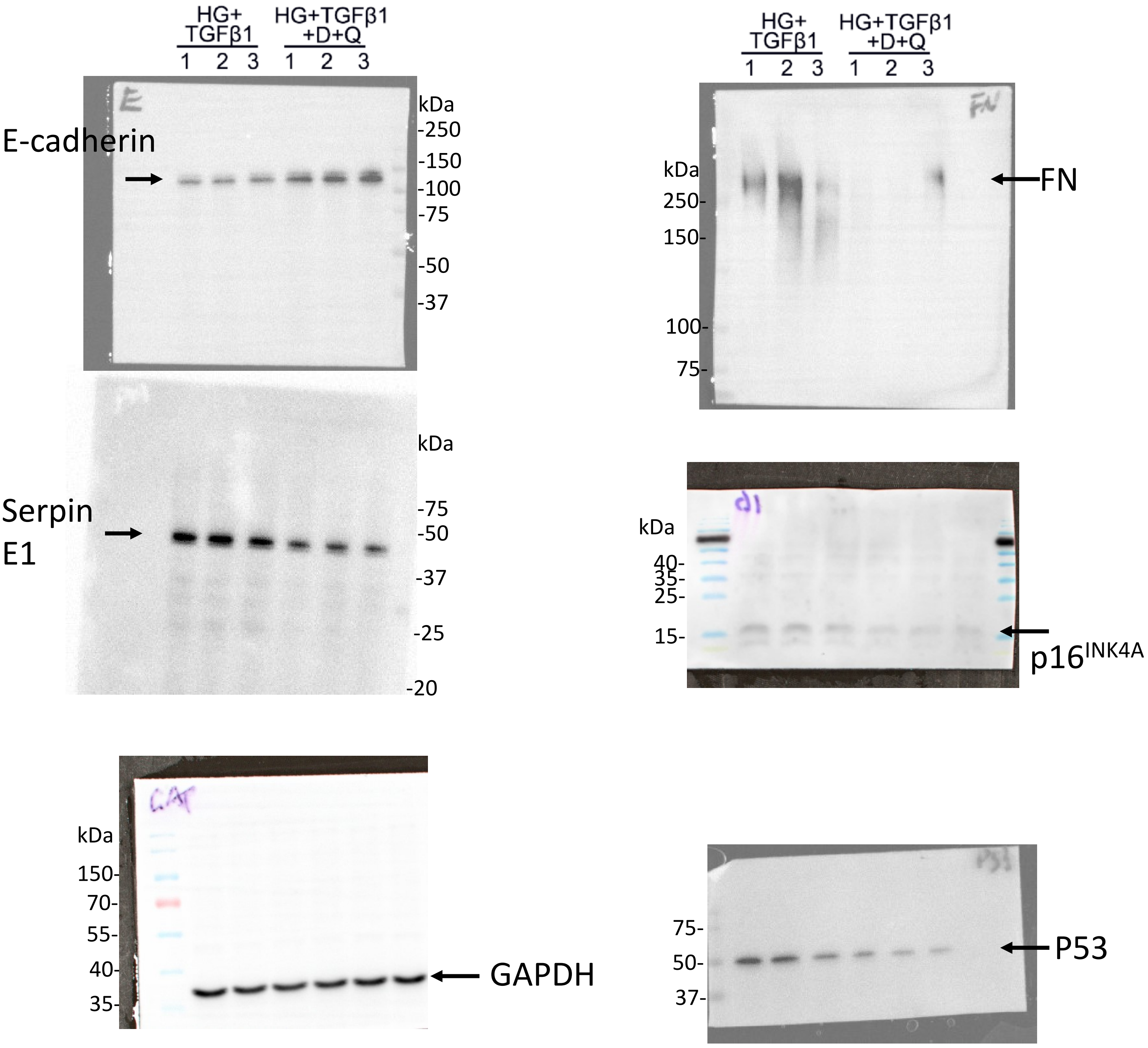

Source Data Figure 2: Uncropped blots used for the construction of composite of Figure 3D.

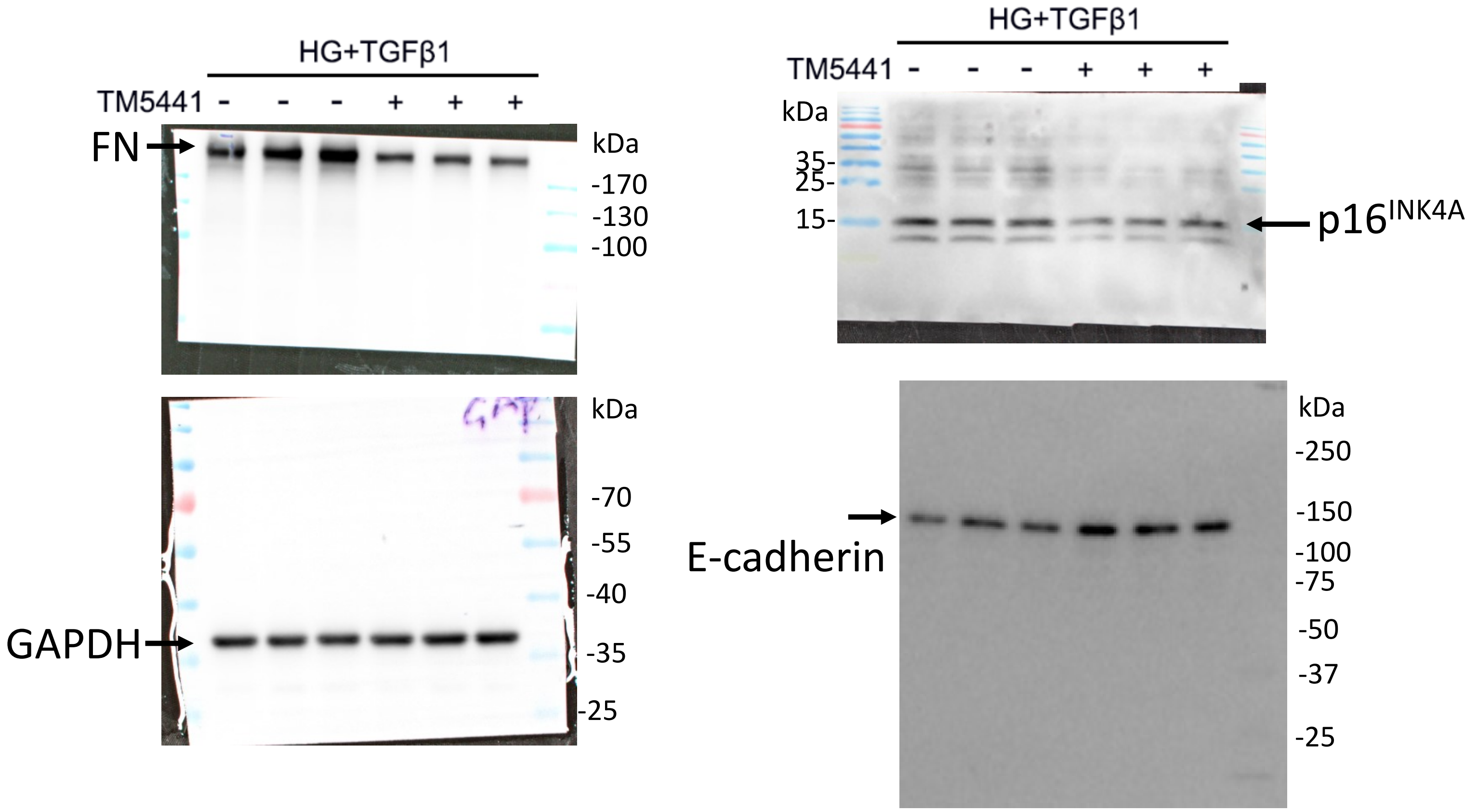

Source Data Figure 3: Uncropped blots used for the construction of composite of Figure 4C.

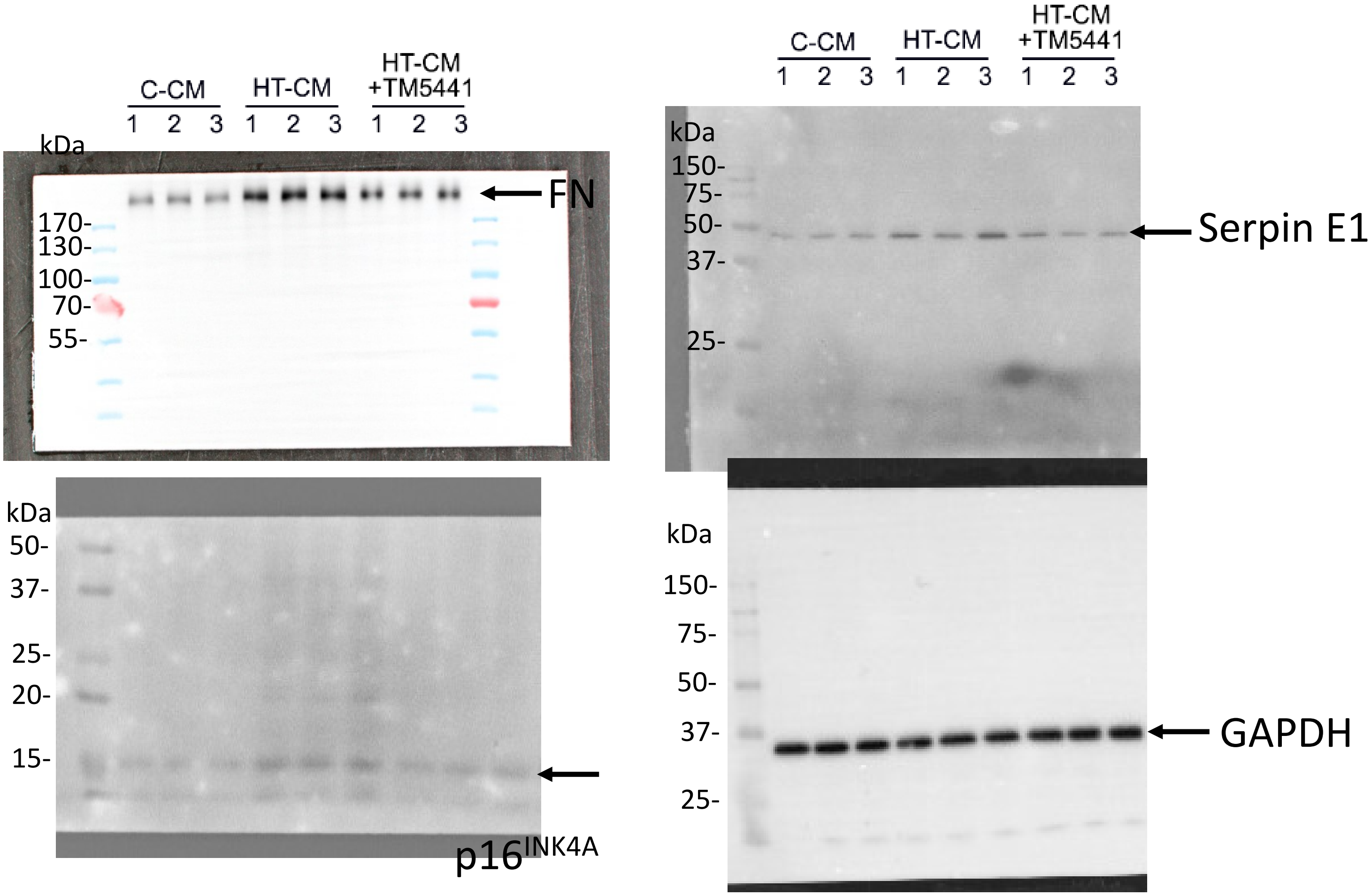

Source Data Figure 4: Uncropped blots used for the construction of composite of Figure 5D.
