# Supplementary material for: Serpin E1 mediates the induction of renal tubular degeneration and premature senescence upon diabetic insult
Source: Sci Rep. 2023 Sep 27;13:16210. doi: 10.1038/s41598-023-43411-4 (PMC10533493; doi:10.1038/s41598-023-43411-4)
Supplement: Supplementary file 2 — Supplementary Information 2. [file 41598_2023_43411_MOESM2_ESM.docx]

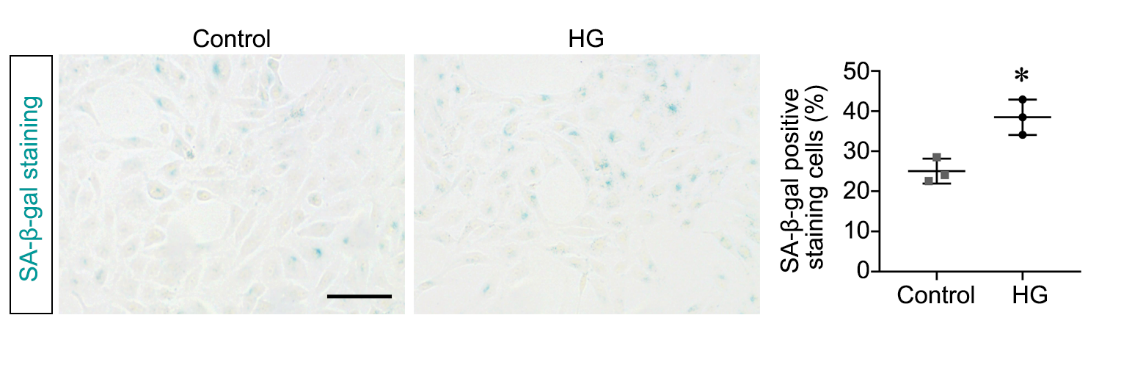


**Supplementary Figure 1. High glucose alone slightly induced tubular cell senescence *in vitro*.** Cultured tubular cells were treated with or without high glucose (HG, 25 mM) for 48h. (A) Cells were processed for SA-β-gal activity staining. Scale bar = 50 μm. (B) Quantification of the SA-β-gal positive cells as percentages of the total number of cells per microscopic field. **P* < 0.05 versus control group (n = 3).

**Supplementary Table 1**. **Demographic, clinical and laboratory data of patients with type 2 diabetic kidney disease or control patients.**

|  | **Control** (n=5) | **DKD** (n=5) | ***P* value** |
| --- | --- | --- | --- |
| ***Demographics*** |  |  |  |
| Age (years) | 55.40±7.83 | 55.00±9.67 | 0.945 |
| Sex (male/female) | 3/2 | 3/2 | >0.999 |
| Hypertension (yes/no) | 4/1 | 2/3 | 0.197 |
| BMI (kg/m^2^) | 24.16±4.59 | 25.11±3.02 | 0.709 |
| ***Laboratory data*** |  |  |  |
| Proteinuria (Yes/no) | 0/5 | 5/0 | 0.002 |
| eGFR (CKD-EPI) (ml/min /1.73m^2^) | 105.9±4.63 | 69.05±33.47 | 0.041 |
| Serum albumin (g/L) | 42.48±2.46 | 43.00 (34.21, 45.90) | 0.841 |
| Serum cholesterol (mmol/L) | 4.01±0.23 | 4.17 (3.00, 6.37) | 0.691 |
| Serum triglyceride (mmol/L) | 2.06±1.03 | 1.64±0.48 | 0.433 |

Abbreviations: BMI, body mass index; DKD, diabetic kidney disease; eGFR, estimated glomerular filtration rate;
