# Supplementary material for: Correction: Serpin E1 mediates the induction of renal tubular degeneration and premature senescence upon diabetic insult
Source: Sci Rep. 2026 Sep 15;16:28728. doi: 10.1038/s41598-026-70468-8 (PMC13578741; doi:10.1038/s41598-026-70468-8)

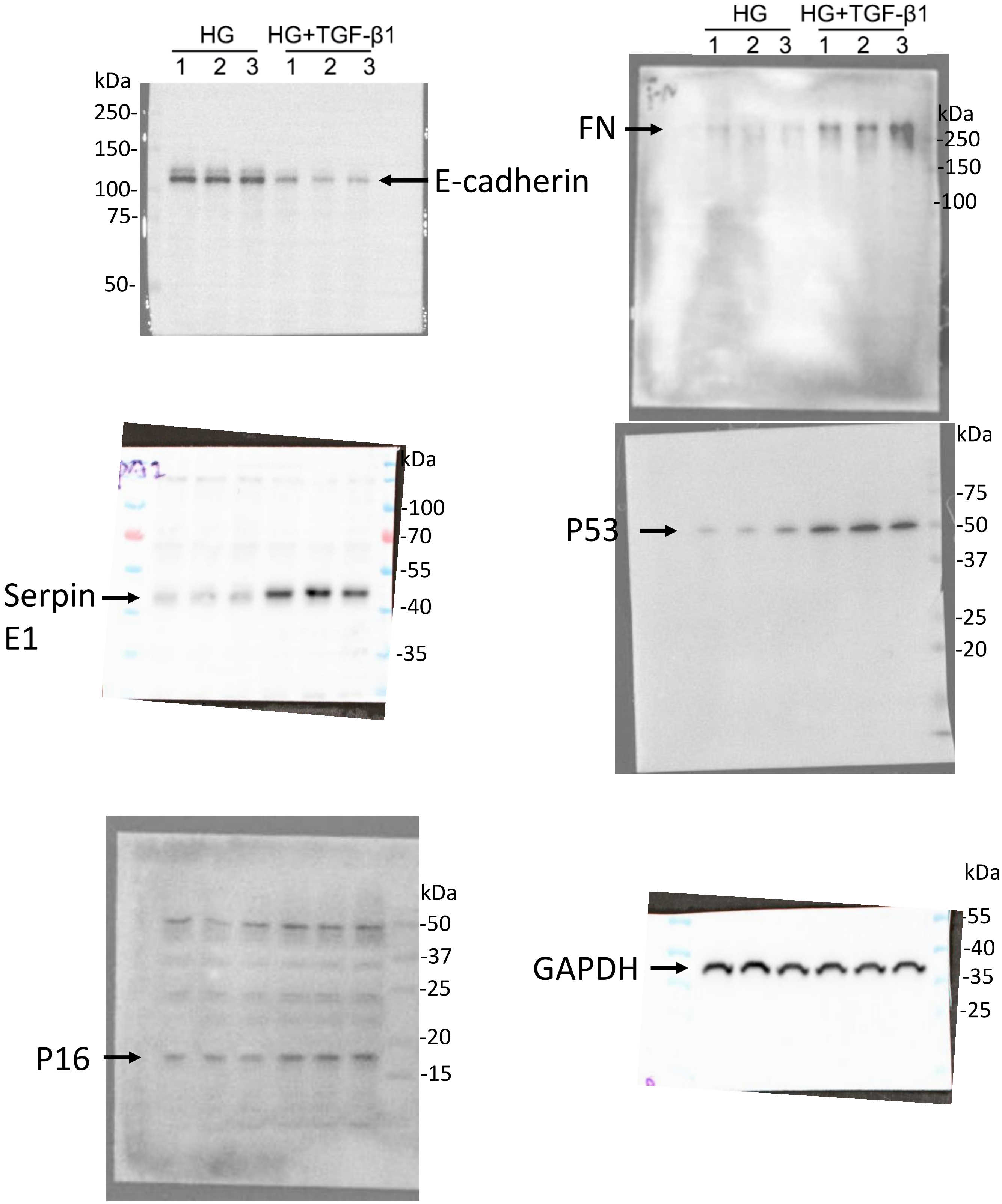

Source Data Figure 1: Uncropped blots used for the construction of composite of Figure 2D.

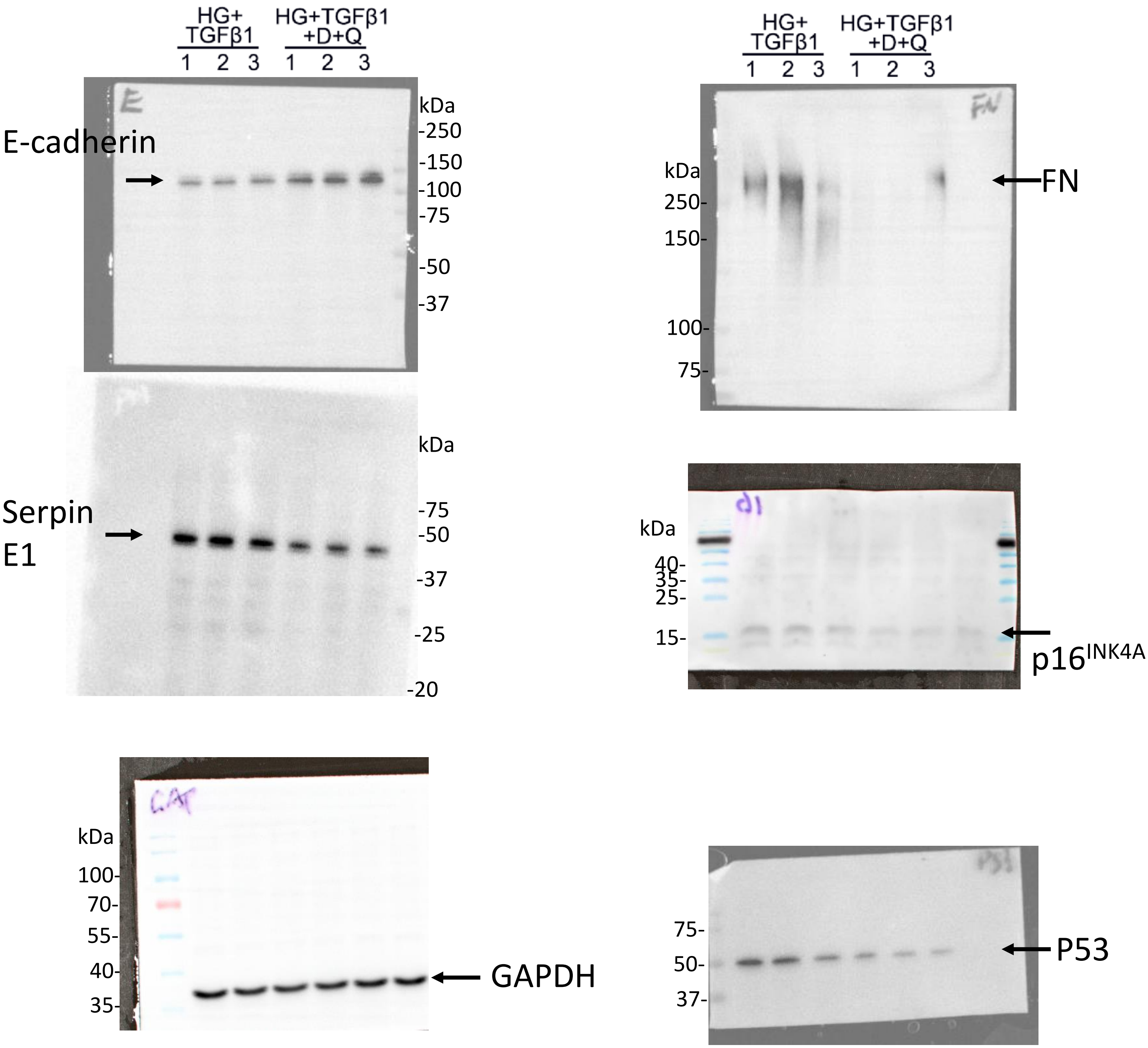

Source Data Figure 2: Uncropped blots used for the construction of composite of Figure 3D.

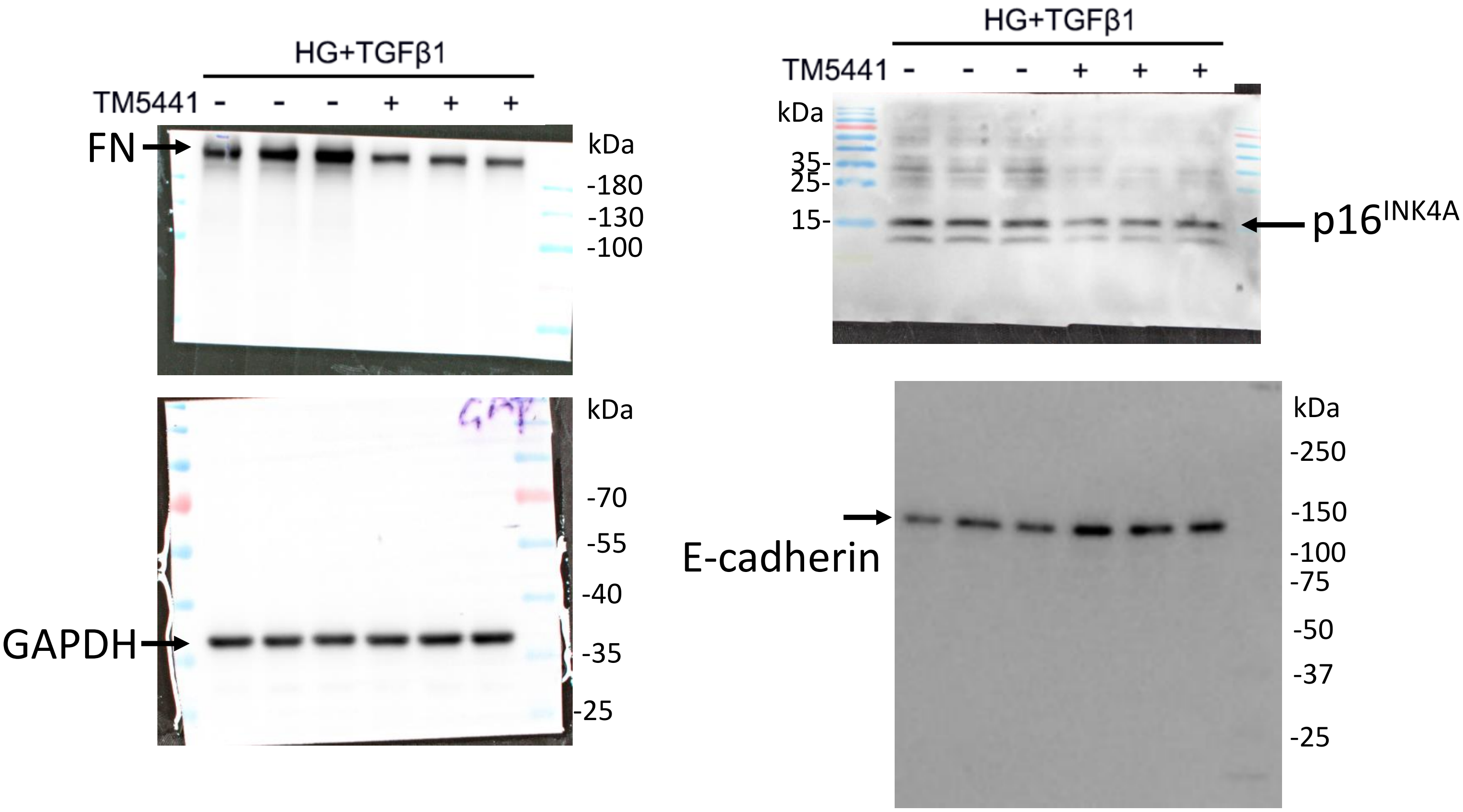

Source Data Figure 3: Uncropped blots used for the construction of composite of Figure 4C.

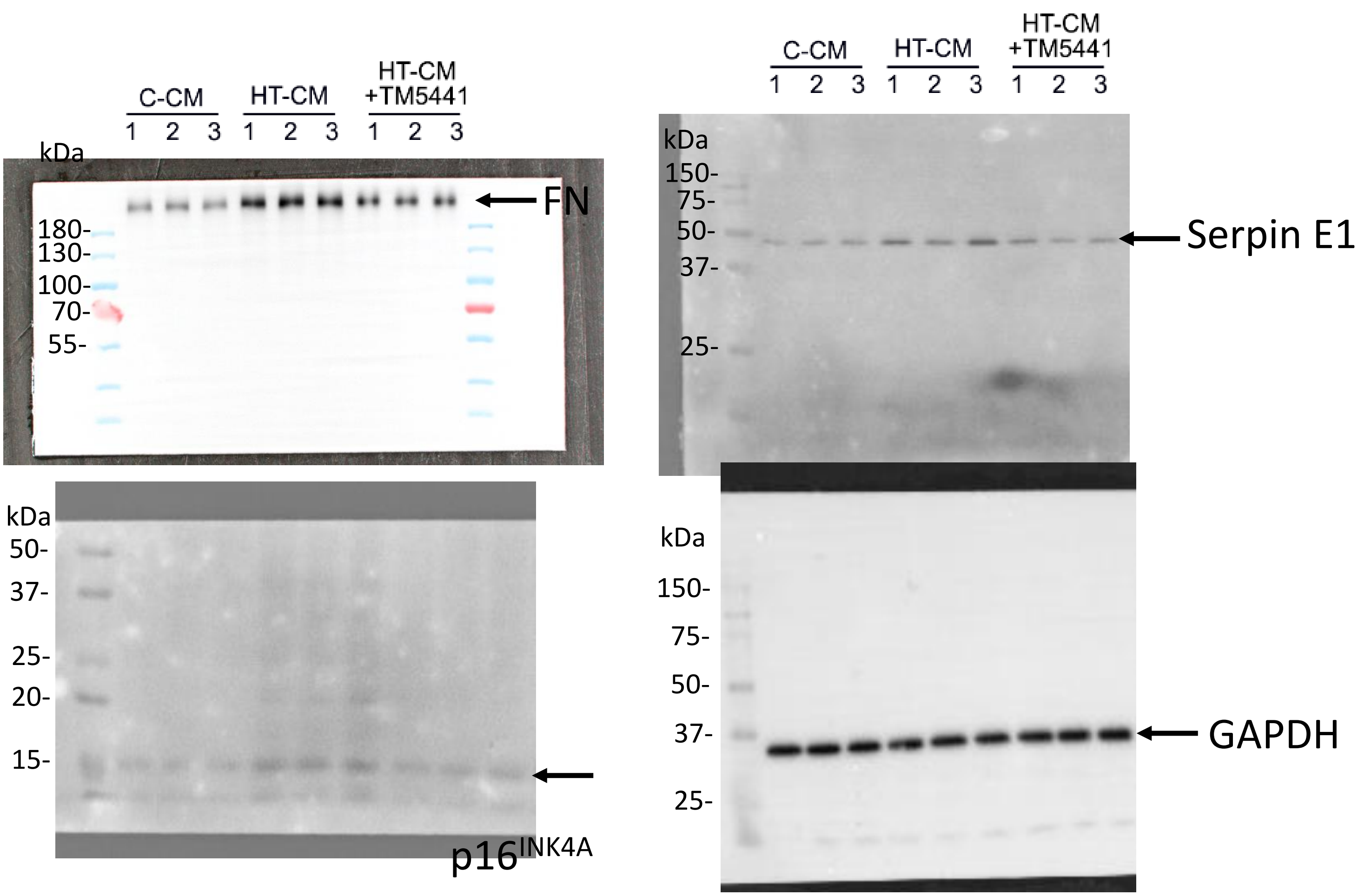

Source Data Figure 4: Uncropped blots used for the construction of composite of Figure 5D.

Two kinds of protein ladders were used in this study. The catalogue numbers are: #1610374 (Biorad) and #26616 (Thermofisher)

#1610374
(Biorad)

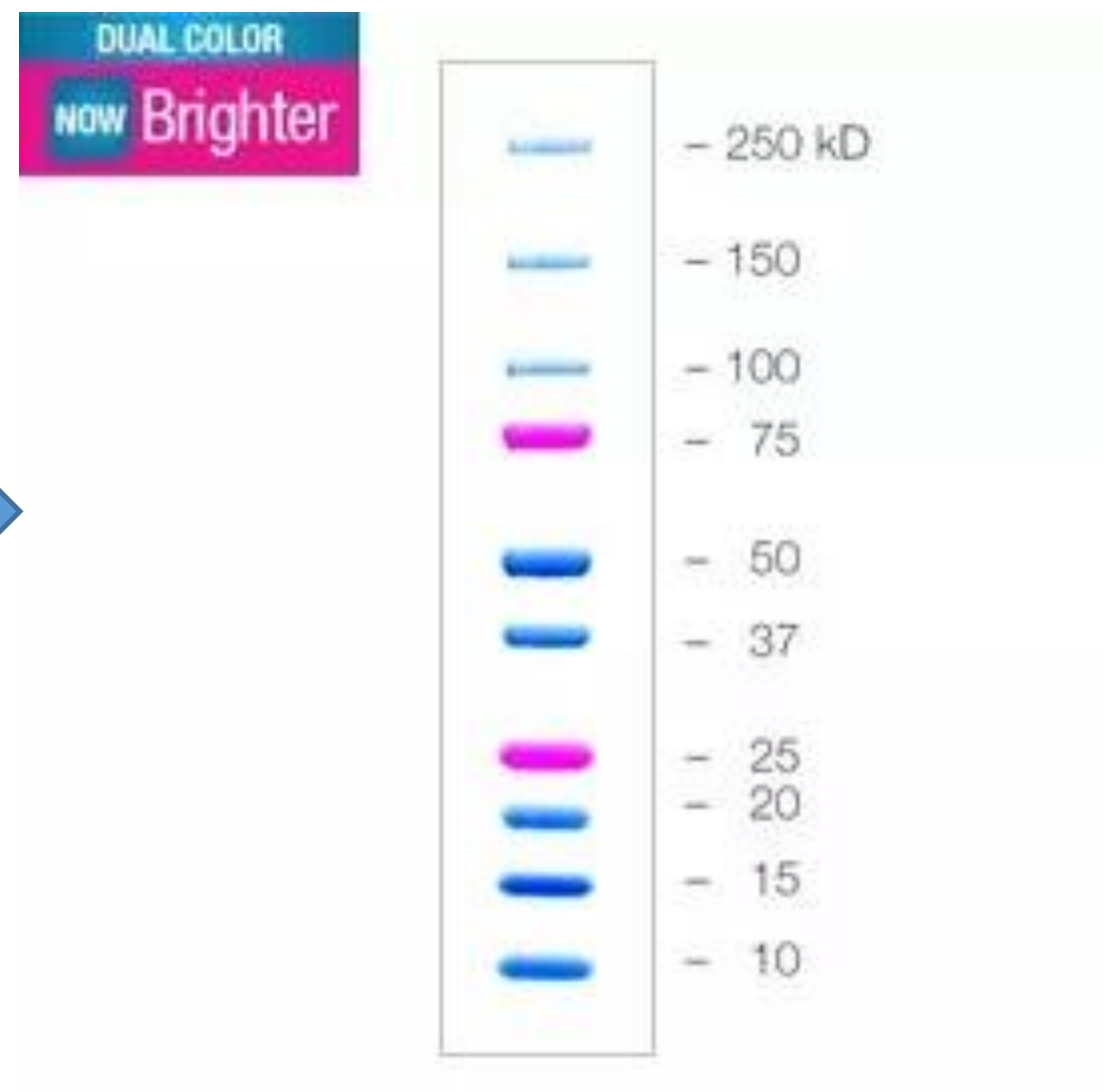

#26616
(Thermofisher)

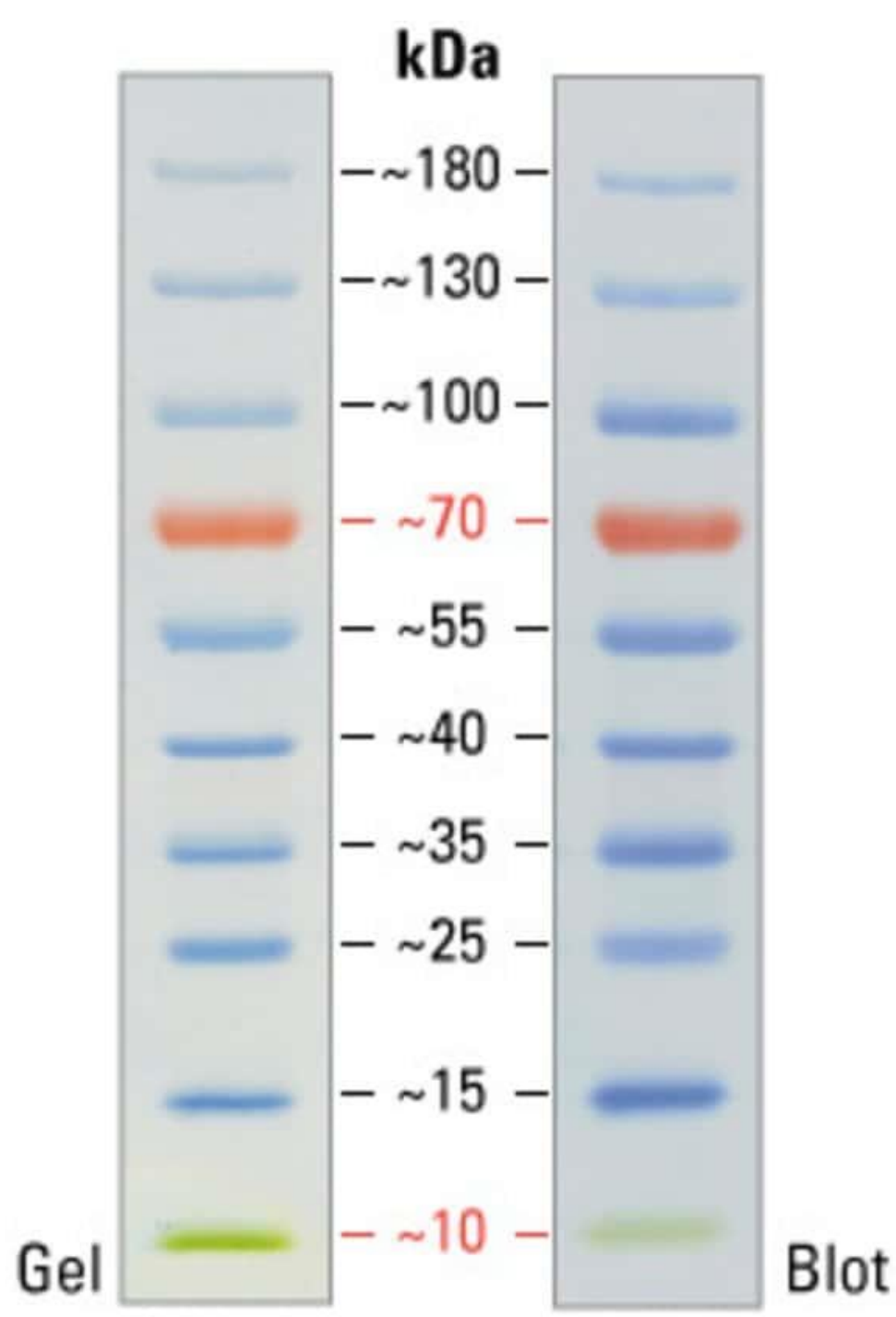

Supplement: Supplementary file 1 [file 41598_2026_70468_MOESM1_ESM.pdf]
